# Elevated TyG index associated with increased prevalence of gallstones in a United States cross-sectional study

**DOI:** 10.3389/fpubh.2024.1351884

**Published:** 2024-05-31

**Authors:** Xueyi Feng, Shenwei Wu, Bin Ke, Yongkang Liang

**Affiliations:** ^1^Department of General Surgery, Lu'an Hospital Affiliated of Anhui Medical University (Lu'an City People’s Hospital), Lu'an, Anhui, China; ^2^Department of Gastrointestinal Surgery, The Second People's Hospital of Wuhu City (Affiliated Wuhu Hospital of East China Normal University), Wuhu, China

**Keywords:** insulin resistance, triglyceride glucose index, gallstones, cross-sectional study, NHANES

## Abstract

**Objective:**

This study aimed to investigate the correlation between the triglyceride-glucose (TyG) index and the incidence of cholelithiasis.

**Research approach:**

In this investigation, a cross-sectional analysis was undertaken utilizing data from the US National Health and Nutrition Examination Survey (NHANES) spanning the years 2017 to 2020. The TyG index served as an independent predictor, while gallstone prevalence was considered the dependent variable of interest. We employed a multivariate logistic regression model to evaluate the interplay between these independent and dependent variables. To assess the presence of potential non-linear associations, sensitivity analysis was executed, utilizing inverse probability weighted validation, smooth curve fitting, and threshold effect analysis. In cases where non-linear relationships were observed, likelihood ratios were utilized to pinpoint potential inflection points. Ultimately, subgroup analyses were conducted to identify specific populations demonstrating heightened susceptibility to gallstone prevalence.

**Results:**

Encompassing 838 patients who self-reported gallstones, a total of 7,794 participants were included in the analytical cohort. A statistically significant disparity in the TyG index was observed when all individuals were categorized into gallstone patients and non-patients (*p* < 0.05). Logistic regression findings indicated a positive correlation between the TyG index and gallstone disease prevalence (OR = 1.28, 95% CI: 1.12, 1.47), with a strengthening association as the TyG index increased (*p* trend <0.01). The results were corroborated by the use of inverse probability weighting. Additionally, a non-linear connection between the TyG index and gallstone prevalence was identified (log-likelihood ratio *p* < 0.01), with the optimal inflection point for TyG calculated at 8.96. In subgroup analysis, the positive relationship between the TyG index and gallstone prevalence was notably pronounced among black Americans under the age of 40 and female participants.

**Conclusion:**

Alterations in the TyG index may potentially correlate with shifts in the prevalence of gallstones among adult populations in the United States. Elevated TyG index values may coincide with an augmented likelihood of gallstone occurrence.

## 1 Introduction

Gallstones (GS) represent one of the most prevalent upper gastrointestinal disorders, manifesting with common symptoms such as abdominal discomfort, upper abdominal pain, nausea, vomiting, and diminished appetite. They affect approximately 20% of the global population (1, 2). Across the world, there are notable variations in the prevalence and formation of gallstones based on race and geographic factors. Approximately 10% of white adults in Western nations experience gallstones (2), while the prevalence stands at around 4% in India (3) and 5.13% in China (4). The incidence of gallstones rises with advancing age, reaching 57% (5). Notably, the United States witnesses over 700,000 cholecystectomies annually, incurring costs of roughly $6.5 billion (6). Extensive efforts have been devoted to unraveling the determinants that elevate the risk of gallstone formation and to develop timely and efficacious preventive strategies.

An established link has been established between gallstones and both insulin resistance and metabolic syndrome (7, 8). Although the hyperinsulinemic normoglycemic clamp (HEC) stands as the gold standard for insulin measurement, it poses practical challenges in non-study settings (9). Given the intricate nature of insulin resistance and the significant time and resource commitments it entails, simpler surrogate markers are frequently utilized for assessment. The TyG index has emerged as a dependable surrogate marker of insulin resistance (1, 10–12). Its utility has been validated across various medical conditions, including cardiovascular disease (13), hearing impairment (11), and proteinuria (10). Nonetheless, its relationship with gallstone prevalence remains unexplored. In summary, this study aimed to investigate the association between the TyG index and the incidence of gallstones within a cross-sectional analysis of the National Health and Nutrition Examination Survey (NHANES) cohort.

## 2 Materials and methods

### 2.1 Study population

**Figure 1:**
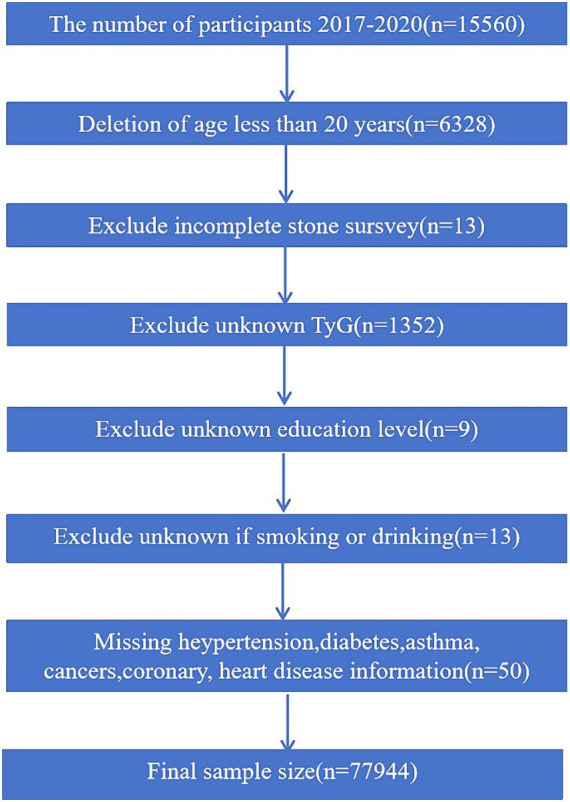
The participants selecting flow chart.

National Health and Nutrition Examination Survey, a recurring cross-sectional survey sponsored by the CDC, has undergone biennial updates for nearly two decades, encompassing approximately 10,000 individuals in each iteration. The NHANES database received approval from the NCHS Institutional Review Board, adhering to the updated Declaration of Helsinki. This investigation utilized data from the 2017 to 2020 timeframe, with participants under the age of 20 being excluded due to the questionnaire’s exclusive administration to adults aged 20 years and older. The study cohort was carefully screened, as delineated in Figure 1, resulting in the inclusion of a total of 7,794 participants.

### 2.2 Data collection and definition

The triglyceride-glucose index (TyG) was formulated as the primary exposure variable. TyG = ln [fasting levels of triglycerides (mg/dL) * fasting plasma glucose (mg/dl)/2]. Triglyceride and fasting plasma glucose levels were quantified employing enzymatic techniques with an automated biochemical analyzer. Serum triglyceride concentrations were assayed utilizing a Roche Cobas 6000 chemistry analyzer in conjunction with a Roche Modular P device.

### 2.3 Assessing diagnosed gallstones

Gallstone status was ascertained through a questionnaire that included the following inquiries: “Have you received a diagnosis of gallstones?” Participants were provided with a choice between two response alternatives: “Affirmative” or “Negative.”

### 2.4 Covariate assessment

Drawing from prior research (14, 15), a multivariate adjusted model was constructed to account for possible confounders. Our analysis incorporated variables such as gender, age, race, educational attainment, poverty-to-income ratio, alcohol consumption, cholesterol levels, uric acid, smoking habits, creatinine levels, history of asthma, hypertension, diabetes, coronary heart disease, and cancer as covariates.

Missing value treatment: in the current study, there were still missing values for BMI, CRP, and PIR. In order to reduce the data bias introduced by the deletion of variables and we interpolated the missing values using the Random Forest method (16), as shown in Supplementary Figure 1.

### 2.5 Statistical methods

Statistical significance was defined as *p* < 0.05. All analyses were performed using the Empower software (www.empowerstats.com; X&Y Solutions, Inc., Boston, MA, United States) and R version 4.0.2 (http://www.R-project.org, The R Foundation). All statistical analyses were conducted employing the NHANES sampling weights, stratification, and clustering specifications provided in the study. To determine the appropriate weights for the variables under study within the largest population, a specific selection of variables was first defined in accordance with the Weit guidelines. In accordance with these guidelines, the study utilized fasting triglyceride data and divided the subweight associated with fasting triglycerides (WTSAF2YR) by 3.2 to derive the final weight (10). Weighted analysis was carried out using the survey design R package in the R programming language. Weighted survey means with 95% confidence intervals were reported for continuous variables, while categorical variables were presented as weighted survey proportions with 95% CIs.

Three distinct multivariate regression models were established (17–19). Model 1 remained unadjusted for covariates, Model 2 was adjusted for sex, age, race, educational attainment, and Model 3 encompassed adjustments for all variables. In the sensitivity analysis, which consists of two parts, the first is the conversion of the TyG variables from a continuous format to two categorical variables in order to assess their robustness. The linear trend was assessed by categorizing TyG into two quintiles. The precision of the outcomes was further evaluated through the utilization of inverse probability weights. Additionally, a generalized additive model (GAM) and smooth curve fitting techniques were applied to account for potential non-linearities in the TyG and gallstone association. In instances where non-linear correlations were identified, a two-segment linear regression model (segmented regression model) was employed to fit each segment and calculate the threshold effect. Second, we found that some previous reports, including METS-IR (8), WWI (20), and VAI (21) indices, were reported to be associated with the prevalence of gallstones, and we adjusted them to further clarify the effect of the TyG index on the prevalence of gallstones. In addition, subgroups were analyzed by gender, age, and ethnicity using stratified multiple regression analysis, and the results were presented using forest plots (22, 23).

## 3 Results

### 3.1 Baseline characteristics

The baseline demographic attributes of the enrolled participants are presented in Table 1. Weighted characteristics were categorized based on gallstone status. Apart from educational level, cholesterol concentrations, and the prevalence of asthma, notable disparities in baseline characteristics were observed between the two cohorts. Those with gallstones exhibited higher age, BMI, CRP levels, and TyG values, a markedly elevated proportion of females, and a greater incidence of medical conditions, including diabetes, hypertension, coronary heart disease, cancer, and asthma.

**Table 1:** Baselines characteristics of participants, weighted.

Characteristic	Stone formers (*n* = 838)	Non-stone formers (*n* = 6,956)	*p* value
Age (years)	58.41 (57.33,59.48)	49.96 (49.53,50.40)	<0.0001
Serum creatinine (mg/dl)	0.92 (0.88,0.97)	0.91 (0.89,0.92)	0.4622
Total bilirubin (mg/dL)	0.46 (0.44,0.48)	0.46 (0.45,0.46)	0.4851
Serum cholesterol (mg/dL)	184.10 (181.24,186.96)	186.30 (185.34,187.27)	0.151
Serum uric acid (mg/dL)	5.49 (5.38,5.59)	5.40 (5.36,5.43)	0.1049
TyG index	8.82 (8.78,8.86)	8.64 (8.63,8.66)	<0.0001
BMI (kg/m^2^)	33.36 (32.77,33.94)	29.65 (29.48,29.83)	<0.0001
PIR	2.55 (2.45,2.66)	2.62 (2.58,2.65)	0.2568
CRP	5.82 (5.14,6.50)	3.99 (3.80,4.19)	<0.0001
Gender (%)			<0.0001
Male	28.53 (25.55,31.71)	50.78 (49.53,52.02)	
Female	71.47 (68.29,74.45)	49.22 (47.98,50.47)	
Race (%)			0.0129
Mexican American	13.52 (11.36,16.01)	11.88 (11.09,12.71)	
White	11.26 (9.30,13.59)	10.20 (9.49,10.96)	
Black	62.15 (58.79,65.40)	60.60 (59.38,61.81)	
Other Race	13.07 (10.95,15.53)	17.32 (16.38,18.30)	
Education level (%)			0.6604
Less than high school	18.54 (16.02,21.36)	18.69 (17.73,19.69)	
High school	25.16 (22.31,28.25)	23.75 (22.72,24.82)	
More than high school	56.29 (52.88,59.65)	57.55 (56.32,58.78)	
Alcohol (%)			<0.0001
Yes	51.73 (48.34,55.10)	40.76 (39.60,41.93)	
No	32.87 (29.78,36.12)	45.17 (43.99,46.37)	
Unclear	15.40 (13.10,18.02)	14.07 (13.26,14.92)	
High blood pressure (%)			<0.0001
Yes	54.92 (51.53,58.26)	36.42 (35.28,37.58)	
No	45.08 (41.74,48.47)	63.58 (62.42,64.72)	
Asthma (%)			0.0001
Yes	20.46 (17.86,23.34)	15.14 (14.32,16.01)	
No	79.54 (76.66,82.14)	84.86 (83.99,85.68)	
Coronary artery disease (%)			<0.0001
Yes	9.00 (7.23,11.15)	4.02 (3.58,4.51)	
No	91.00 (88.85,92.77)	95.98 (95.49,96.42)	
Cancers (%)			<0.0001
Yes	17.74 (15.29,20.50)	9.48 (8.82,10.19)	
No	82.26 (79.50,84.71)	90.52 (89.81,91.18)	
Diabetes (%)			<0.0001
Yes	26.24 (23.36,29.33)	13.91 (13.11,14.76)	
No	73.76 (70.67,76.64)	86.09 (85.24,86.89)	
Smoked (%)			0.0094
Yes	45.94 (42.57,49.35)	41.25 (40.08,42.43)	
No	54.06 (50.65,57.43)	58.75 (57.57,59.92)	

Data of continuous variables are shown as survey-weighted mean (95%CI), *p* value was calculated by survey-weighted linear regression. Data of categorical variables are shown as survey-weighted percentage (95%CI), *p* value was calculated by survey-weighted Chi-square test.

### 3.2 Higher prevalence of gallstones associated with higher TyG index

**Figure 2:**
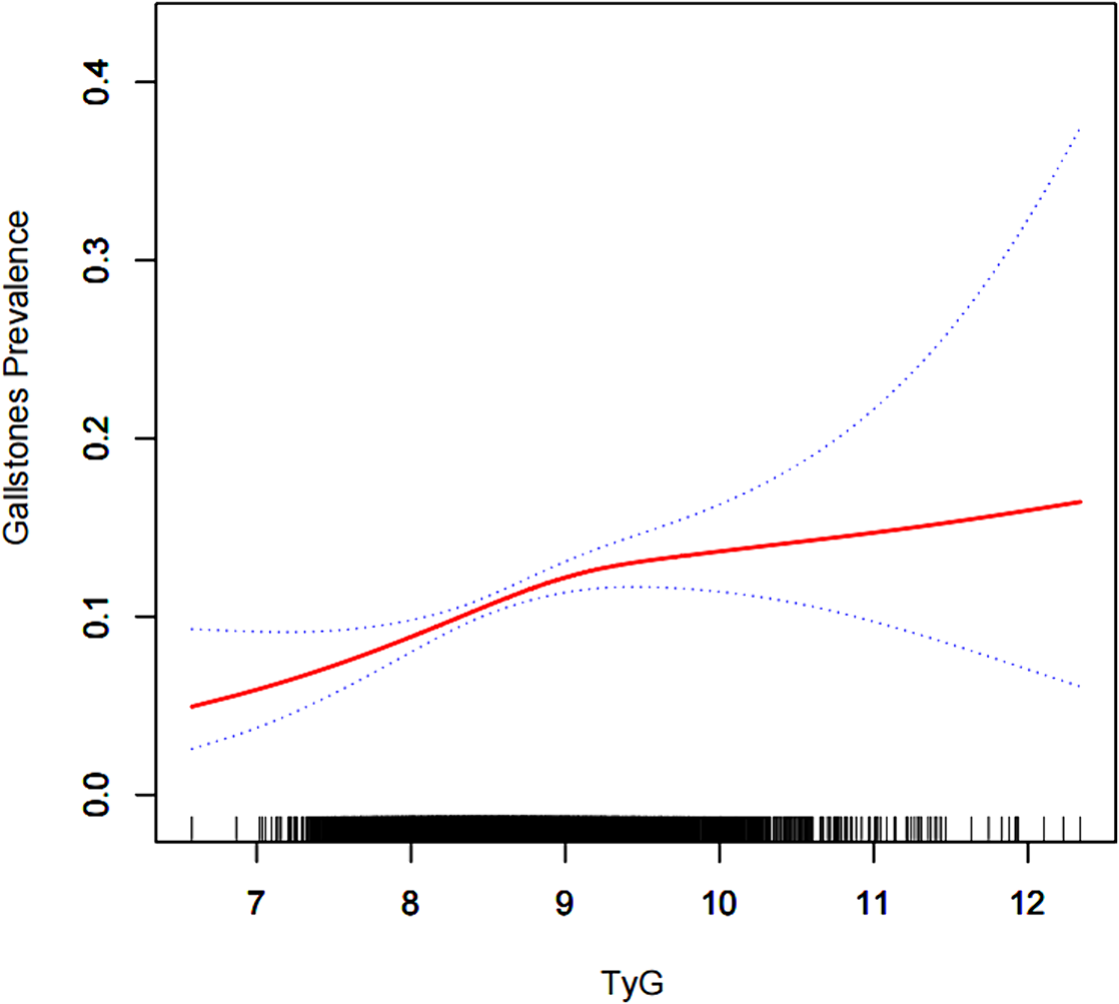
Density dose–response relationship between TyG index with gallstone prevalence. The area between the upper and lower dashed lines is represented as 95% CI. Each point shows the magnitude of the TyG index and is connected to form a continuous line. Adjusted for all covariates except effect modifier.

In the fully adjusted model, a one-unit increment in the TyG index was associated with a 28% elevated risk of gallstones (OR = 1.28, 95% CI: 1.12, 1.47). The effect of TyG index on gallbladder stones remained significant even after adjusting for the METS-IR, VAI, and WWI indices (Supplementary Table 1). Upon dichotomizing the TyG index, logistic regression revealed a noteworthy 36% escalated risk of gallstones in the highest TyG index group compared to the lowest (OR = 1.36, 95% CI: 1.15, 1.61, *p* for trend <0.01). In addition, inverse probability weighted analysis results were computed based on TyG two-score. Supplementary Table 2 demonstrates that the baseline characteristics were essentially balanced between the two groups, and the results of inverse probability weighted logistic regression indicated a 36% increased risk of gallstones in the highest TyG index group compared to the lowest (OR = 1.36, 95% CI: 1.12, 1.65). Smooth curve fitting was subsequently employed to explore the association between the TyG index and gallstone prevalence. Our findings revealed a non-linear positive correlation between the TyG index and gallstone prevalence (Figure 2). A similar likelihood ratio test identified a threshold effect for the TyG index and gallstone prevalence, with the optimal inflection point determined as 8.96 (Tables 2, 3).

**Table 2:** Logistic regression analysis between TyG index with gallstones prevalence.

Characteristic	Model 1 OR (95%CI)	Model 2 OR (95%CI)	Model 3 OR (95%CI)	Model 4 OR (95%CI)
TyG	1.48 (1.33, 1.64)	1.47 (1.31, 1.65)	1.28 (1.12, 1.47)	-
Categories				
Lower (6.58–8.60)	1	1	1	1
Higher (8.60–12.34)	1.73 (1.49, 2.01)	1.60 (1.37, 1.87)	1.36 (1.15, 1.61)	1.36 (1.12, 1.65)★
*p* for trend	<0.01	<0.01	<0.01	<0.01

Model 1 was adjusted for no covariates; Model 2 was adjusted for age, gender, race, and education; Model 3 was adjusted for covariates in Model 2 + diabetes, blood pressure, PIR, smoked, physical activity, alcohol use, serum cholesterol, TBIL,CRP, uric acid serum creatinine, coronary artery disease, asthma, and cancers were adjusted. Model 4 adjusts the same covariates as model 3; ^★^IPTW analysis only in model 4.

**Table 3:** Two-piecewise linear regression and logarithmic likelihood ratio test explained the threshold effect analysis of TyG index with gallstones prevalence.

TyG index	ULR test	PLR test	LRT test
	OR (95%CI)	OR (95%CI)	*p* value
<8.96	1.28 (1.12, 1.47)	1.65 (1.30, 2.11)	<0.0001
≥8.96		0.99 (0.77, 1.27)	

ULR, Univariate linear regression; PLR, Piecewise linear regression; LRT, Logarithmic likelihood ratio test, statistically significant: *p* < 0.05.

### 3.3 Subgroup analysis

**Figure 3:**
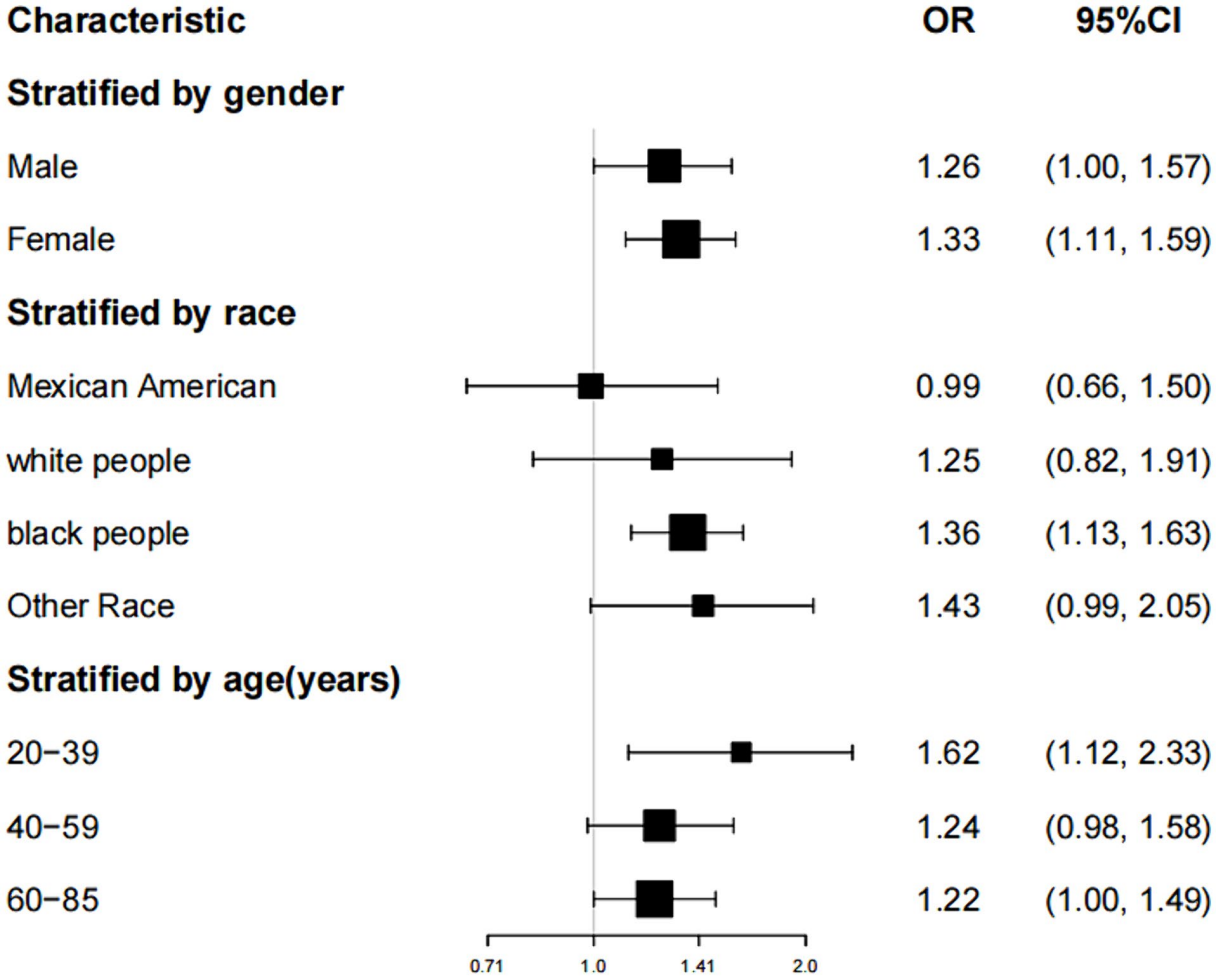
Forest plot of subgroup analysis between TyG index and gallstone prevalence in model 3.

Subgroup analyses were conducted to evaluate the resilience of the association between the TyG index and the prevalence of gallstones. Findings (Table 4; Figure 3): Male subgroup (OR = 1.26, 95% CI: 1.00, 1.57), Female subgroup (OR = 1.33, 95% CI: 1.11, 1.59), Age < 40 years subgroup (OR = 1.62, 95% CI: 1.12, 2.33), Age 40–59 years subgroup (OR = 1.24, 95% CI: 0.98, 1.58), Age 60 years subgroup (OR = 1.22, 95% CI: 1.00, 1.49), Mexican-American subgroup (OR = 0.99, 95% CI: 0.66, 1.50), White subgroup (OR = 1.25, 95% CI: 0.82, 1.91), Black subgroup (OR = 1.36, 95% CI: 1.13, 1.63), and Other ethnicity subgroup (OR = 1.43, 95% CI: 0.99, 2.05).

**Table 4:** Subgroup regression analysis between TyG index with gallstones prevalence.

Characteristic	Model 1 OR (95%CI)	Model 2 OR (95%CI)	Model 3 OR (95%CI)
Stratified by gender			
Male	1.38 (1.16, 1.65)	1.32 (1.09, 1.60)	1.26 (1.00, 1.57)
Female	1.88 (1.64, 2.16)	1.62 (1.39, 1.88)	1.33 (1.11, 1.59)
Stratified by race			
Mexican American	1.06 (0.80, 1.40)	1.05 (0.75, 1.48)	0.99 (0.66, 1.50)
White people	1.30 (0.97, 1.75)	1.24 (0.88, 1.74)	1.25 (0.82, 1.91)
Black people	1.64 (1.43, 1.88)	1.62 (1.40, 1.89)	1.36 (1.13, 1.63)
Other Race	1.63 (1.23, 2.14)	1.55 (1.15, 2.10)	1.43 (0.99, 2.05)
Stratified by age(years)			
20–39	1.53 (1.19, 1.97)	2.25 (1.69, 2.99)	1.62 (1.12, 2.33)
40–59	1.31 (1.10, 1.57)	1.53 (1.27, 1.86)	1.24 (0.98, 1.58)
60–85	1.29 (1.10, 1.52)	1.33 (1.12, 1.57)	1.22 (1.00, 1.49)

Model 1 was adjusted for no covariates. Model 2 was adjusted for age, gender, race, and education; Mode3 = adjusted for all covariates except effect modifier.

## 4 Discussion

Our examination demonstrated a notable and robust correlation between the TyG index and the prevalence of gallstones, even following comprehensive adjustments for pertinent confounding factors in the fully adjusted model. Additional curve fitting and assessment of threshold effects disclosed a non-linear interrelationship between the TyG index and gallstone prevalence, pinpointing a threshold at 8.96. Consequently, the monitoring of the TyG index in patients could serve as a straightforward and efficient instrument in epidemiological investigations concerning gallstones.

The global prevalence of cholelithiasis is substantial, impacting around 5–25% of adults, particularly within the Western world (24). In the United States alone, the burden of gallstone-related conditions results in approximately 1.5 million physician visits (25). Given that cholelithiasis is a chronic ailment entailing morbidity, diminishing quality of life, and considerable healthcare expenses, its prevention assumes paramount significance. The existing pressures have witnessed continued escalation across the globe. Effective strategies for gallstone prevention can be enhanced by identifying an appropriate population for the TyG index. Therefore, we conducted a subgroup analysis, revealing that within the age-stratified analysis, the TyG index exerted its most pronounced influence on gallstone prevalence in the age group under 40 years. The role of age as a risk factor for cholelithiasis remains a subject of debate. An Italian study identified advancing age as a risk factor for gallstone incidence (26), whereas a Taiwanese study associated age with gallbladder and fatty liver disease risk (27). Conversely, some investigations have posited that metabolic syndrome and obesity have a more substantial impact on gallstones in younger individuals (28), and a separate NHANES study by Wang et al. concurs with our findings (8). Concerning gender, the data suggest that female patients may bear a higher risk of gallstones (2, 29), a conclusion supported by our results as well. In the United States, black Americans exhibit the highest prevalence of cholelithiasis, which could be linked to dietary and lifestyle disparities among various ethnic groups. A significant majority of black Americans are employed for fewer hours compared to their white counterparts, often leading to high-calorie dietary choices, identified as a risk factor for gallstones (6). This is a plausible explanation for the elevated incidence of gallstones observed in this demographic.

In our investigation, we employed a comprehensive regression model to mitigate the impact of potential confounding factors, enhance result stability, and elucidate the relationship between the TyG index and gallstone prevalence. We implemented inverse probability weighting (IPTW) to harmonize patient characteristics across both groups (30). IPTW has been explored using a progressively escalating approach (31). Equilibrating baseline data differences permits a more effective reduction of confounding influences on the findings. In our research, as variable disparities gradually diminished post-IPTW, the influence of the TyG index on gallstone prevalence remained consistent, further substantiating result stability. In addition, to illustrate the independent effect of TyG on the prevalence of gallstones, we included the insulin resistance index METS-IR, and we also included the visceral obesity indices WWI and VAI for adjustment, and then excluded the effects of abdominal obesity and insulin resistance, the effect of TyG index on the prevalence of gallstones still existed, which indirectly suggests that an increase in the TyG index is closely related to an increase in the prevalence of gallstones and can be used as a predictor of the prevalence of gallstones. However, due to the drawbacks of cross-sectional studies, a multicenter prospective cohort study is necessary.

The precise mechanism linking the heightened TyG index and the increased prevalence of gallstones remains uncertain, yet previous research offers insights into potential mechanisms. Investigations have demonstrated that insulin resistance in high-risk Hispanic individuals can result in cholesterol-saturated bile, disrupting gallbladder function and precipitating gallstone formation (7). Animal studies have indicated that mice with isolated hepatic insulin resistance (LIRKO mice) have an increased propensity to develop cholesterol gallstones (32). Additionally, in mouse models, a high-protein, high-quality diet has been associated with accelerated bile acid and gallstone formation (33). Another contributory factor in gallstone pathogenesis is leptin, often linked to hyperleptinemia in cases of insulin resistance (34). *In vivo* experiments have verified that prolonged intraperitoneal administration of high-dose leptin (10 μg/g per day) induces weight loss and cholesterol gallstone formation in C57BL/6 J ob/ob mice (35). Furthermore, *in vitro* experiments have demonstrated that leptin impacts gallstone formation by regulating bile acid metabolism (36).

Our study possesses several key strengths. Firstly, NHANES, with its representative United States sample, enforced strict adherence to a meticulously crafted study protocol, including robust quality control and assurance measures, thus underpinning the reliability of our findings. Secondly, NHANES contributed a wealth of demographic and metabolic data, complemented by an extensive follow-up spanning more than 20 years. This enabled comprehensive adjustments for the primary confounding factors within our multivariate model. We further mitigated the influence of confounding variables and broadened the applicability of our results through subgroup analyses. Nevertheless, our study does present certain limitations. Initially, it was a cross-sectional investigation, precluding the establishment of causal relationships between TyG and gallstones. Additionally, the reliance on questionnaire-based survey data in NHANES introduces the potential for recall bias. Despite these constraints, our study represents the first exploration of the association between TyG and gallstone prevalence and provides compelling evidence for the utility of TyG as a gallstone development predictor.

## 5 Summary

This investigation indicates a connection between increased TyG levels and an elevated risk of gallstone prevalence, with notable advantages potentially more pronounced in younger adults. Nonetheless, the confirmation of our results warrants further examination through additional research.

## Data availability statement

The datasets presented in this study can be found in online repositories. The names of the repository/repositories and accession number(s) can be found in the article/Supplementary material.

## Ethics statement

The studies involving humans were approved by the NCHS Research Ethics Review Committee approved the NHANES survey protocol (https://www.cdc.gov/nchs/nhanes/irba98.htm), and all participants of the study provided informed written consent. The NHANES database is open to the public and therefore the ethical review of this study was exempt. The studies were conducted in accordance with the local legislation and institutional requirements. The participants provided their written informed consent to participate in this study. Written informed consent was obtained from the individual(s) for the publication of any potentially identifiable images or data included in this article.

## Author contributions

XF: Conceptualization, Data curation, Investigation, Writing – original draft. SW: Methodology, Software, Writing – original draft. BK: Formal analysis, Visualization, Writing – original draft. YL: Writing – review & editing.
