# Supplementary figures and images for: Elevated TyG index associated with increased prevalence of gallstones in a United States cross-sectional study
Source: Front Public Health. 2024 May 31;12:1351884. doi: 10.3389/fpubh.2024.1351884 (PMC11177685; doi:10.3389/fpubh.2024.1351884)

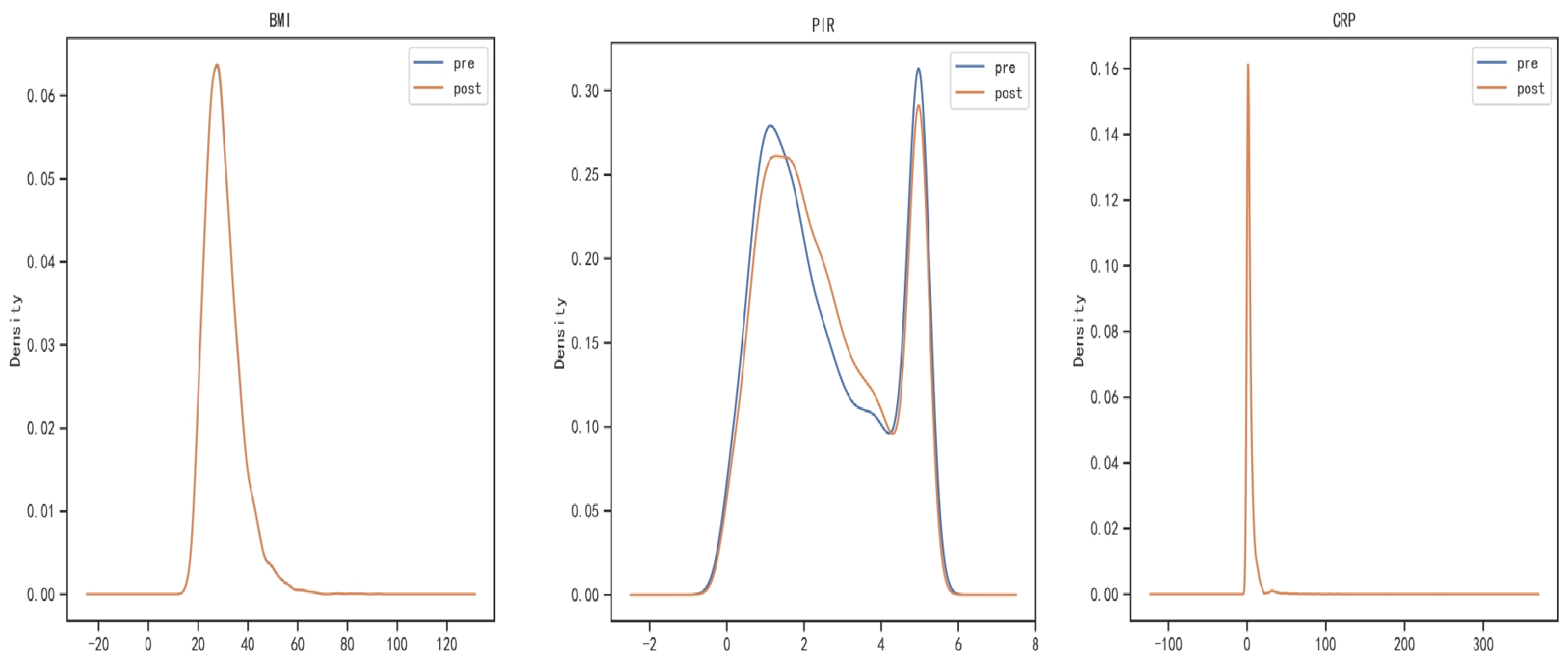

Supplement: Supplementary file 1 [file Image_1.TIF]
