# Supplementary material for: Elevated TyG index associated with increased prevalence of gallstones in a United States cross-sectional study
Source: Front Public Health. 2024 May 31;12:1351884. doi: 10.3389/fpubh.2024.1351884 (PMC11177685; doi:10.3389/fpubh.2024.1351884)
Supplement: Supplementary file 2 [file Table_1.docx]

Supplementary table 1. Logistic regression analysis between TyG index with gallstones prevalence

| **Characteristic** | **Model 1 OR(95%CI)** | **Model 2 OR(95%CI)** | **Model 3 OR(95%CI)** | **Model 4 OR(95%CI)** |
| --- | --- | --- | --- | --- |
| TyG | 1.48 (1.33, 1.64) | 1.47 (1.31, 1.65) | 1.24(1.01, 1.51) | 1.66 (1.01, 2.47) |

Model 1 was adjusted for no covariates;

Model 2 was adjusted for age,gender,race and education;

Model 3 was adjusted for covariates in Model 2+diabetes,blood pressure, PIR, smoked, physical activity, alcohol use, serum cholesterol, TBIL,CRP, uric acid serum creatinine, coronary artery disease, asthma, cancers, WWI and VAI were adjusted.

Model4 was adjusted for covariates in Model 3+METS-IR

Supplementary Table 1.Comparison of baseline data for asthma duration classification by using IPTW.

| Variables | Lower | Higher | P value |
| --- | --- | --- | --- |
| Age(years) | 52.00 ± 17.65 | 51.88 ± 16.93 | 0.7644 |
| Serum Creatinine(mg/dl) | 0.94 ± 0.47 | 0.92 ± 0.69 | 0.2674 |
| Total Bilirubin(mg/dl) | 0.46 ± 0.27 | 0.46 ± 0.31 | 0.3242 |
| Serum Cholesterol(mg/dl) | 188.49 ± 47.43 | 186.56 ± 40.44 | 0.0539 |
| Serum Uric Acid(mg/dl) | 5.57 ± 1.65 | 5.46 ± 1.46 | 0.0512 |
| BMI(kg/m2) | 30.97 ± 9.50 | 30.39 ± 6.84 | 0.062 |
| PIR | 2.60 ± 1.52 | 2.64 ± 1.55 | 0.3354 |
| CRP | 5.43 ± 11.58 | 4.32 ± 8.34 | <0.0001 |
| Gender(%) |  |  | 0.3639 |
| Male | 0.483 | 0.493 |  |
| Female | 0.517 | 0.507 |  |
| Race(%) |  |  | 0.6124 |
| Mexican American | 0.116 | 0.122 |  |
| White | 0.103 | 0.103 |  |
| Black | 0.619 | 0.606 |  |
| Other Race | 0.162 | 0.169 |  |
| Education Level(%) |  |  | 0.8325 |
| Less than high school | 0.181 | 0.186 |  |
| High school | 0.235 | 0.234 |  |
| More than high school | 0.584 | 0.58 |  |
| Alcohol(%) |  |  | 0.5975 |
| Yes | 0.419 | 0.425 |  |
| No | 0.444 | 0.433 |  |
| Unclear | 0.137 | 0.142 |  |
| High Blood Pressure(%) |  |  | 0.2758 |
| Yes | 0.412 | 0.4 |  |
| No | 0.588 | 0.6 |  |
| Asthma(%) |  |  | 0.7783 |
| Yes | 0.158 | 0.155 |  |
| No | 0.842 | 0.845 |  |
| Coronary Artery Disease(%) |  |  | 0.9503 |
| Yes | 0.047 | 0.046 |  |
| No | 0.953 | 0.954 |  |
| Cancers(%) |  |  | 0.6065 |
| Yes | 0.108 | 0.105 |  |
| No | 0.892 | 0.895 |  |
| Diabetes(%) |  |  | 0.0715 |
| Yes | 0.165 | 0.158 |  |
| No | 0.845 | 0.842 |  |
| Smoked(%) |  |  | 0.2417 |
| Yes | 0.434 | 0.421 |  |
| No | 0.566 | 0.579 |  |

P-value were from weighted t test for continuous variables, and weighted chi-square test for categorical variables

For continuous variables, Standardized difference = abs(Mean1-Mean0)/sqrt((S1+S2)/2)

For categorical variables, Standardized difference = abs(P1-P0)/sqrt((P1*(1-P1)+P0*(1-P0))/2)

Supplementary Figure1.Data interpolation for BMI,PIR and CRP using random forest approach.
